# Epidemiological and comparative genomic analysis of pathogenic *Glaesserella parasuis* from livestock agriculture in Shandong, China

**DOI:** 10.3389/fmicb.2025.1698342

**Published:** 2025-10-08

**Authors:** Zetong Huang, Qinghai Ren, Xusheng Du, Shengliang Cao, Yubao Li

**Affiliations:** ^1^Phage Research Center of Liaocheng University, Liaocheng, China; ^2^School of Agriculture and Biology, Liaocheng University, Liaocheng, Shandong, China; ^3^School of Pharmacy and Food Engineering, Liaocheng University, Liaocheng, Shandong, China; ^4^Shandong Key Laboratory of Applied Technology for Protein and Peptide Drugs, School of Pharmaceutical Sciences and Food Engineering, Liaocheng University, Liaocheng, China

**Keywords:** *Glaesserella parasuis*, epidemiology, whole genome sequencing, antimicrobial resistance, comparative genomics

## Abstract

*Glaesserella parasuis* (*G. parasuis*) is the causative agent of Glässer’s disease, posing a significant economic threat to the livestock agriculture. The present study aimed to investigate the prevalence of *G. parasuis* in six regions of Shandong Province, China, from June 2023 to November 2024, and to analyze the whole genome of *G. parasuis* isolates using whole genome sequencing (WGS). This study conducted a comprehensive analysis of the isolates, encompassing antibiotic resistance profiling, virulence gene detection, multilocus sequence typing (MLST), prophage detection, and pan-genome analysis. The prevalence of *G. parasuis* ranged from 10.8 to 26.5% across different cities in Shandong Province, exhibiting significant seasonal variation (*p* < 0.01). Among the 45 isolates, serovar 4 accounted for the highest proportion (40%). Antibiotic resistance testing revealed that 55.6% of isolates demonstrated multidrug-resistance (MDR). WGS analysis revealed that 18 distinct sequence types (STs) were identified across the 45 isolates, including 13 newly discovered STs. Notably, all isolates possessed complete prophage sequences. Pan-genome and phylogenetic analysis of 145 *G. parasuis* strains indicated that *G. parasuis* possesses an open pan-genome with genetic diversity. In summary, these data enhance our understanding of the molecular characteristics and epidemiological risks of *G. parasuis* in Shandong Province, China, particularly regarding livestock agriculture.

## 1 Introduction

In recent years, livestock agriculture in swine has made enormous progress (Warr et al., 2020). Nevertheless, the spread of bacterial and viral diseases has worsened owing to intensive swine agriculture. Among the major bacterial pathogens affecting swine, *Glaesserella parasuis* (*G. parasuis*) plays an important role. Therefore, studying the epidemiology and genomics of *G. parasuis* on swine farms will contribute to the sustainable development of livestock agriculture.

*G. parasuis* is a strictly nicotinamide adenine dinucleotide (NAD)-dependent gram-negative bacterium that causes fibrinous polyserositis, arthritis, fibrinous pleurisy, and meningitis in swine, leading to significant economic losses in livestock agriculture worldwide (Fu et al., 2018). *G. parasuis* is a commensal organism of the upper respiratory tract, but under appropriate conditions can invade and cause severe systemic disease. This disease is primarily observed to break out when the immune system of swine is immature or is affected by other stressors (An et al., 2023). Currently, 15 serovars and a considerable number of non-typable *G. parasuis* strains have been identified (Angen et al., 2004; Howell et al., 2015). Antimicrobial treatment is a crucial method for controlling Glässer’s disease. These include aminoglycosides, amphenicols, fluoroquinolones, lincosamides, macrolides, sulfonamides, tetracyclines, and *β*-lactams, which have been used to control this disease in swine through feed, water, or injection (Dayao et al., 2016; González-Fernández et al., 2024). However, the irregulated use of antimicrobials is considered the main reason for the accumulation of antimicrobial resistance (AMR) (Zhou et al., 2010). The AMR and antimicrobial resistance genes (ARGs) of *G. parasuis* vary in different regions (Ge et al., 2024; González-Fernández et al., 2024). The pathogenicity of *G. parasuis* depends on a variety of virulence factors acting synergistically. Key among these are surface structures crucial for adhesion and immune evasion, including the outer membrane protein *ompP2* (Lee et al., 2023), capsular polysaccharides synthesized by proteins like *capD* to resist phagocytosis (Wang et al., 2013), and surface-modifying sialyltransferases encoded by *lsgB* for immune camouflage (Wang et al., 2018). Concurrently, *G. parasuis* secretes factors that damage host tissues, such as effector proteins delivered via *vta* autotransporters (Olvera et al., 2012) and the cytolethal distending toxin (*cdt*) (Zhou et al., 2012).

As genome research continues, approximately 908 *G. parasuis* genomes are preserved in the National Center for Biotechnology Information (NCBI) database (accessed on June 26, 2025). Since the first publicly available genome sequence of *G. parasuis* was disclosed in 2011 (Mullins et al., 2011), whole-genome sequencing (WGS) has significantly contributed to our deeper understanding of *G. parasuis*. This technology is rapidly emerging as a benchmark in research on bacterial diseases (Mustafa, 2024). WGS has been used for bacterial subtyping (Klemm and Dougan, 2016), pan-genomic analysis (Caputo et al., 2019), among other things. WGS is a crucial instrument for examining a variety of pathogenic microorganisms. It has been widely utilized to investigate the origin, evolution, and spread of numerous important pathogens, such as *Escherichia coli* (Subhadra et al., 2018) and *Salmonella typhimurium* (Bloomfield et al., 2017), among others.

Presently, Shandong Province is an important region for livestock agriculture in China, with a large number of swine breeding facilities. Understanding the pathogenicity profile and genomic nuances of cultured *G. parasuis* is critical for deciphering its molecular signature. This study documented in detail the prevalence of *G. parasuis* across six regions in Shandong Province, China, and conducted a comparative analysis of the genome sequences of 45 *G. parasuis* isolates extracted from diseased swine tissues. Species identification and multi-locus sequence typing (MLST) analysis were conducted on *G. parasuis*, along with the characterization of prophage sequences found in the genomes of 45 *G. parasuis* isolates. In addition, we analyzed the correlation between ARGs and AMR phenotypes, and performed pan-genomic and phylogenetic analyses based on the core genome. This study lays the foundation for future exploration of the molecular epidemiology and potential mechanisms of the pathogenicity of *G. parasuis*.

## 2 Materials and methods

### 2.1 Source and serovar identification of *Glaesserella parasuis* isolates

The study involved the use of 45 pathogenic *G. parasuis* isolates from swine farms and slaughterhouses in Shandong Province, China, including Liaocheng, Dezhou, Weifang, Qingdao, Jining, and Linyi. These isolates were collected between June 2023 and November 2024. They were obtained by inserting sterile loops into tissue samples from lesions, such as lung tissue from deceased swine, or by swabbing the noses of affected swine. The collected samples were streaked on tryptic soy agar plates (TSA; Thermo Fisher Scientific Co., Ltd.) containing 5 μg/mL NAD (Beijing Solarbio Science & Technology Co., Ltd.) and 5% newborn calf serum (Beijing Solarbio Science & Technology Co., Ltd.), and incubated at 37 °C for 24–48 h. Translucent colonies, which had a diameter of 1 mm, were transferred to fresh plates and grown under identical conditions. Bacterial species were identified using 16S rRNA diagnostic polymerase chain reaction (PCR) (Oliveira et al., 2001). The resulting bacterial cultures were freeze-dried and kept at −80 °C. Supplementary Table S1 provides an overview of 45 *G. parasuis* isolates. The serovar of the isolate was determined by conventional PCR using primers listed in Supplementary Table S2 (Howell et al., 2015; Jia et al., 2017).

### 2.2 Antimicrobial susceptibility test

All *G. parasuis* isolates were tested for 15 antibiotics, including cefotaxime (30 μg), amoxicillin (20 μg), ampicillin (10 μg), cefalexin (30 μg), amikacin (30 μg), gentamycin (10 μg), neomycin (30 μg), tetracycline (30 μg), erythromycin (15 μg), ciprofloxacin (5 μg), enrofloxacin (10 μg), levofloxacin (5 μg), florfenicol (30 μg), cotrimoxazole (1.25/23.75 μg), and clindamycin (2 μg) were assessed using the Kirby-Bauer disk diffusion susceptibility testing technique^1^ on antibacterial tablets (Hangzhou Microbial Reagent Co., Ltd. Hangzhou, Zhejiang, China). It is crucial for these isolates to comply with the recommendations set forth by the Clinical Laboratory Standards Institute (CLSI: https://clsi.org/). Based on the CLSI criteria for interpreting zone diameters, each strain was categorized as resistant, intermediate, or susceptible (Humphries et al., 2021). *H. influenzae* ATCC 49247 was used as the quality control strain. Strains that are resistant to at least three different classes of antibiotics, excluding cross-resistance mechanisms, are classified as multidrug-resistant (MDR) (Magiorakos et al., 2012).

### 2.3 Amplification of virulence genes and ARGs

Basic PCR tests were performed to identify the presence of 26 ARGs and 14 virulence genes. The following ARGs were identified: sulfonamides (*sul1*, *sul2* and *sul3*), tetracyclines (*tetA*, *tetB* and *tetC*), macrolides (*erm(A)*, *erm(B)* and *erm(C)*), fluoroquinolones (*gyrA*, *gyrB*, *parC* and *parE*), aminoglycosides (*aacC2*, *aadB*, *aacC4*, *aphA1*, *strA* and *strB*), amphenicols (*floR*), *β*-lactams (*tem*, *shv*, *ctx* and *dha*), and trimethoprims (*dfrA3* and *dfrA-1-15-16*). The identified virulence genes included those encoding proteins involved in a sialyltransferase involved in lipooligosaccharide synthesis (*lsgB*), capsule synthesis and export (*capD*, *wza*), trimeric autotransporters (*vta1*, *vta2* and *vta3*), hemolysin activation (*hhdA*, *hhdB*), outer membrane integrity maintenance (*ompP2*), oxidative tolerance association (*nanH*), cytolethal distending toxin (*cdt*), and extracellular serine protease (*espP2*). Supplementary Table S3 (De Gheldre et al., 2003; Lanz et al., 2003; Matter et al., 2007) and Supplementary Table S4 (Sack and Baltes, 2009; Zhang et al., 2013) present a comprehensive list of ARGs and virulence genes, accompanied by their respective primer sequences and the size of the amplification products. The PCR mixture had a total volume of 25 μL, and include 12.5 μL of 2 × Taq Master Mix (Nanjing Vazyme Biotech Co., Ltd.), 1 μL of each primer, 8.5 μL double distilled H_2_O, and 2 μL of gDNA for each strain. All PCR assays were performed using a A300 Gradient PCR Instrument (Hangzhou LongGene Scientific Instrument Co., Ltd., China), and the cycling conditions were optimized for each target gene (Supplementary Tables S3, S4). PCR products were confirmed by 1% agarose gel electrophoresis and visualized under UV light.

### 2.4 Genome sequencing assembly, and annotation

Genomic DNA from *G. parasuis* was extracted using a TIANamp Bacteria DNA Kit (Tengen Biochemical Technology Co., Ltd.). Whole-genome sequencing of the 45 *G. parasuis* isolates was conducted by Novogene Technology Co., Ltd. (Beijing, China) using the Illumina Novaseq-PE150 platform. In short, all data were filtered using fastp v0.23.4 (Chen et al., 2018) and fastqc.v0.12.1 to remove adapters and low-quality reads. SPAdes v3.13.1^2^ (Bankevich et al., 2012) was used to splice the genome sequences and filter out fragments below 200 bp. The completeness of the genome assembly was then assessed using CheckM v1.2.3^3^ (Donovan et al., 2015). The NCBI for Biotechnology Information Prokaryotic Genome Annotation Pipeline (PGAP v6.4) was utilized to carry out the genome annotation (Tatusova et al., 2016). The sequencing data were then used to perform statistical analyses and make subsequent genetic predictions.

### 2.5 *Glaesserella parasuis* MLST analysis, and prophage prediction

The genomes of the 45 *G. parasuis* isolates were submitted to the PubMLST *G. parasuis* database (https://pubmlst.org/organisms/glaesserella-parasuis, accessed on March 2025) (Jolley et al., 2018) for MLST analysis. The allele and sequence type (ST) associated with each MLST gene were identified by comparison with the allele and ST present in the database. Data for 860 STs were obtained from the PubMLST *G. parasuis* database (accessed on March 22, 2025). Strain ST clustering was analyzed using goeBURST v1.2.1 (Francisco et al., 2009) software under the triple-site variants (TLVs) criterion. Single nucleotide polymorphisms (SNPs) were identified using CSI Phylogeny 1.4^4^ (Kaas et al., 2014), and a genetic evolutionary tree was constructed based on high-quality comparisons of SNPs. Final visualization of the tree was performed using iTOL v6^5^ (Letunic and Bork, 2024). PHASTER^6^ (Arndt et al., 2016) software was used to identify prophage loci within the *G. parasuis* genome and analyze prophage sequence features. The PHASTEST score was based on the number of coding sequences (CDSs) present in the DNA sequence and the presence or absence of phage-associated genes. The predicted prophage genome data will be collated and analyzed in terms of genome size and GC content.

### 2.6 *Glaesserella parasuis* pan-genome analysis

Phylogenetic connections between various strains of *G. parasuis* have been investigated by retrieving the complete genomes of 100 *G. parasuis* strains in China that were accessible through the NCBI database (excluding repetitive genome sequences, download date: March 2025) (Supplementary Table S5). The script *bp_genbank2gff3.pl* was utilized to create GFF3 files for 145 strains, which served as input for Roary v3.13.0 (Page et al., 2015). The pan-genome curve fitting is given by *N = an ^-b^*, where *N* represents the total number of genes, *n* indicates the number of genomes, and a and b are the parameters used for fitting. In the case where b < 1, the curve fitting expands with the addition of new strains and is designated as “open.” Conversely, when b > 1, the curve fitting will be “closed.” Python was utilized to execute *roary_plots.py*, a supplementary procedure to the Roary software, to demonstrate the percentage of diverse taxonomic genes and the number of strains. The optimal model was determined using ModelFinder (Kalyaanamoorthy et al., 2017) in IQ-TREE v1.6 (Nguyen et al., 2014), based on the core genome sequences. The maximum likelihood estimation of 1,000 bootstrap replications was then used to construct the genetic evolution tree, which was visualized using iTOL v6^7^ (Letunic and Bork, 2021).

### 2.7 Statistical analysis

All statistical analyses were performed using software SPSS v28.0 (IBM Corporation, United States) and GraphPad Prism v9.0 (GraphPad Software Inc., San Diego, CA, United States). All tests were two-sided, and differences were considered to be highly significant if *p* < 0.01, or significant if *p* < 0.05. The relationship between ARGs and AMR phenotypes was evaluated using the kappa coefficient. These were interpreted as follows: < 0: no concordance; 0.00–0.20: slight concordance; 0.21–0.40: fair concordance; 0.41–0.60: moderate concordance; 0.61–0.80: substantial concordance; and ≥ 0.8: excellent concordance (Landis and Koch, 1977; Liou et al., 2011).

## 3 Results

### 3.1 Prevalence and distribution of *Glaesserella parasuis* in Shandong Province, China

**Figure 1:**
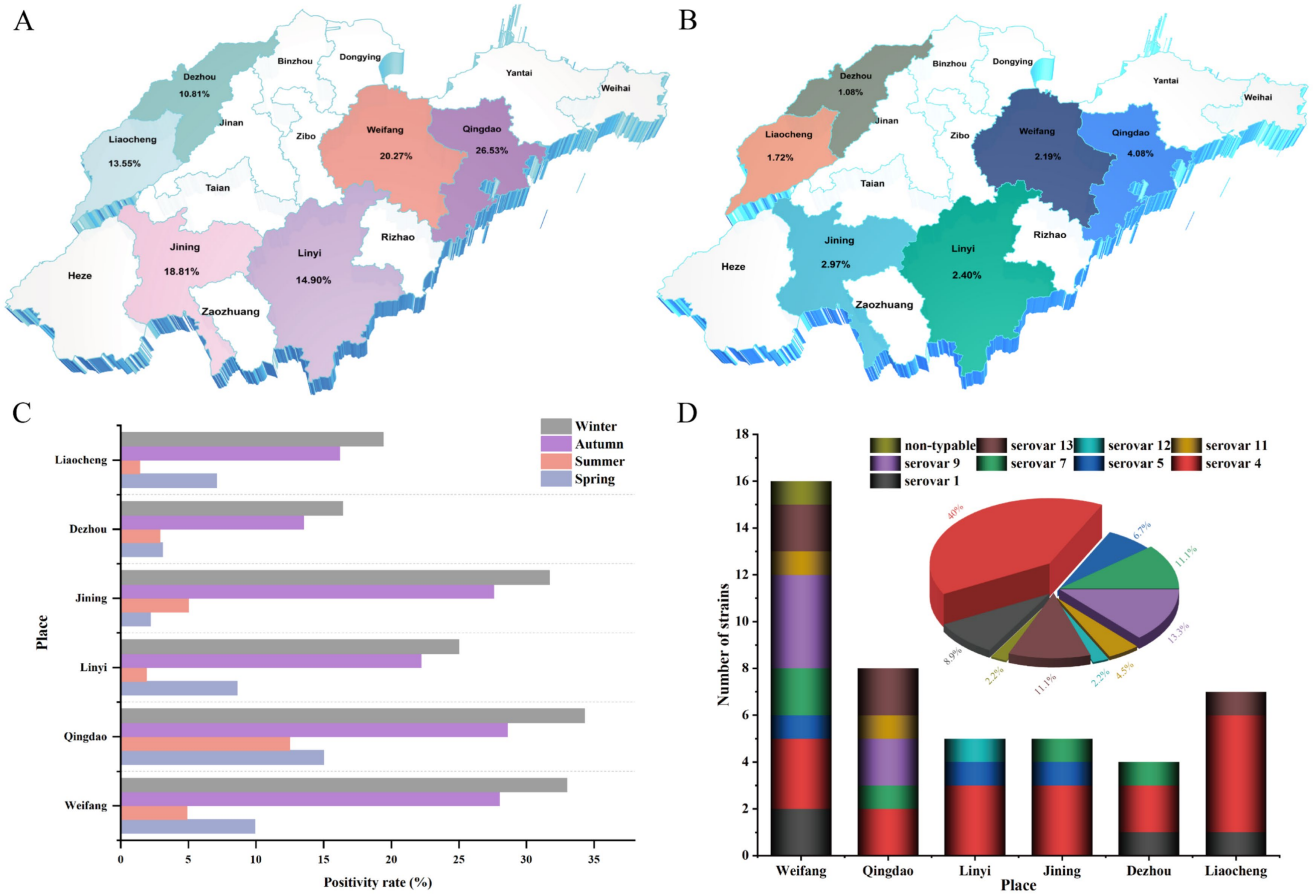
Geographic distribution and epidemiological overview of in Shandong Province, China. **(A)** Regional distribution of *G. parasuis* infections. **(B)** Isolation of *G. parasuis* isolates from different regions. **(C)** The distribution of *G. parasuis* infections in different regions and seasons. **(D)** The serovar distribution of *G. parasuis* isolates from different regions.

In this study, varying degrees of respiratory symptoms and typical Glässer’s disease were observed in intensive swine farms (Supplementary Figure S1). All isolates formed colonies with similar morphology on TSA plates, the colonies were circular, with smooth, moist surfaces and entire edges (Supplementary Figure S2). A total of 2,112 samples were collected from swine farms and slaughterhouses across six regions in Shandong Province from June 2023 to November 2024. The results showed that the total positive rate of the samples was 17.2% (364/2112) and the total isolation rate was 2.1% (45/2112) (Supplementary Table S5). The positivity rate across different regions ranged from 10.8% (40/370) to 26.5% (52/196) (Figure 1A), whereas the isolation rate varied from 1.1% (4/370) to 4.1% (8/196) (Figure 1B). Among these, the highest positivity (26.5%) and isolation (4.1%) rates were observed in Qingdao. Notably, the positivity rate of *G. parasuis* during the autumn and winter seasons was significantly higher than that in the spring and summer in various regions (*p* < 0.01) (Figure 1C). Among the 45 *G. parasuis* isolates, serovar 4 accounted for the highest proportion (40.0%), followed by serovar 9 (13.3%). Various serovars were isolated from different regions, with the strains from Weifang exhibiting the greatest diversity, isolating a total of eight serovars: serovar 1, 4, 5, 7, 9, 11, and 13, and one isolate that could not be classified into a specific serovar. The distribution of the strains was primarily concentrated in Weifang (*n* = 16) (Figure 1D).

### 3.2 Analysis of ARGs and AMR phenotypes

**Figure 2:**
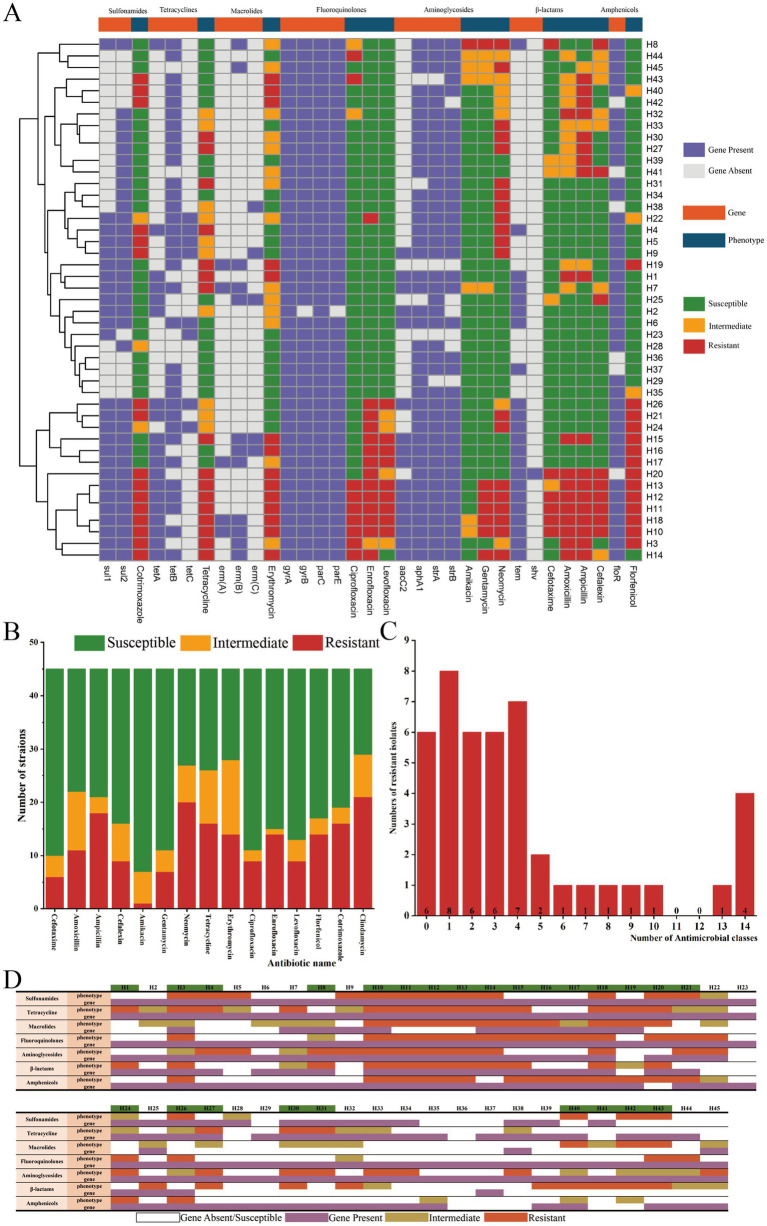
Results of *G. parasuis* resistance analysis. **(A)** AMR phenotypes and identification of ARGs. **(B)** Number of strains susceptible, moderately susceptible, and resistant to antibiotics. **(C)** Numbers of resistant *G. parasuis* isolates. **(D)** Correlation of antimicrobial phenotypes with ARGs in 45 *G. parasuis* isolates from Shandong provinces, China, with green color markers representing MDR strains.

Among the 26 antibiotic resistance genes detected, all strains carried at least seven resistance genes, wherein the fluoroquinolone resistance genes *gyrA* and *parC* were most prevalent (100%) (Figure 2A). Among the aminoglycoside resistance genes, *strA* had the highest detection rate (41/45, 91.1%), whereas *aadB* and *aacC4* were not detected. *TetB* had the highest detection rate among tetracycline resistance genes (33/45, 73.3%). *Sul2* had the highest detection rate among the sulfonamide resistance genes (35/45, 77.8%), whereas *sul3* was not detected. *FloR* identification rate was 86.67% (39/45). *Tem* was detected in 48.89% (22/45) of *β*-lactam resistance genes, but *ctx* and *dha* were not detected. The lowest detection rate of macrolide resistance genes was observed in the overall results, with the highest *erm(B)* detection rate of 26.67% (12/45). Notably, no trimethoprim resistance genes (*dfrA3* and *dfrA-1-15-16*) were identified in any of the isolates. We identified 21 isolates exhibiting a high level of resistance to lincomycin (Figure 2A). From the overall results of the resistance phenotype, *G. parasuis* strains exhibited a high degree of resistance to clindamycin and neomycin, with resistance rates of 46.7% (21/45) and 44.4% (20/45), respectively. Conversely, the strains demonstrated the lowest resistance to amikacin, with a rate of 2.2% (1/45) (Figure 2B). We also found that 25 isolates were classified as multidrug-resistant (MDR) by mediating resistance to several antibiotics of different classes (Figure 2C). Moreover, we observed that the *G. parasuis* isolate from Linyi showed a higher resistance than the isolate from Weifang (*p* < 0.05). Analysis of the correlation between ARGs and AMR phenotypes revealed that macrolides showed the highest concordance rate (30/45, 66.7%), followed by β-lactams (27/45, 60.0%) and aminoglycosides (22/45, 48.9%). Of note, except for fluoroquinolones (kappa = 0), all other drug classes showed slight agreement, with kappa values ranging from 0.0120–0.2068 (Figure 2D; Table 1).

**Table 1:** Kappa consistency test for ARGs and AMR phenotypes.

Class	TP	FP	FN	TN	Kappa	Cn	Agreement
Sulfonamides	13	23	3	6	0.0120	42.2%	Slight
Tetracycline	16	25	0	4	0.1022	44.4%	Slight
Macrolides	6	8	7	24	0.2068	66.7%	Fair
Fluoroquinolones	17	28	0	0	0	37.8%	No
Aminoglycosides	20	23	0	2	0.0717	48.9%	Slight
β-lactams	12	10	8	15	0.1980	60.0%	Slight
Amphenicols	13	26	1	5	0.0603	40.0%	Slight

TP (True Positive): both resistant genes and drug resistance, FP (False Positive): resistant genes but no drug resistance, FN (False Negative): resistant but no resistant genes, TN (True Negative) neither resistant genes nor drug resistance, Cn (degree of conformity): (TP+TN)/total number of strains × 100%.

### 3.3 Analysis of virulence genes

**Figure 3:**
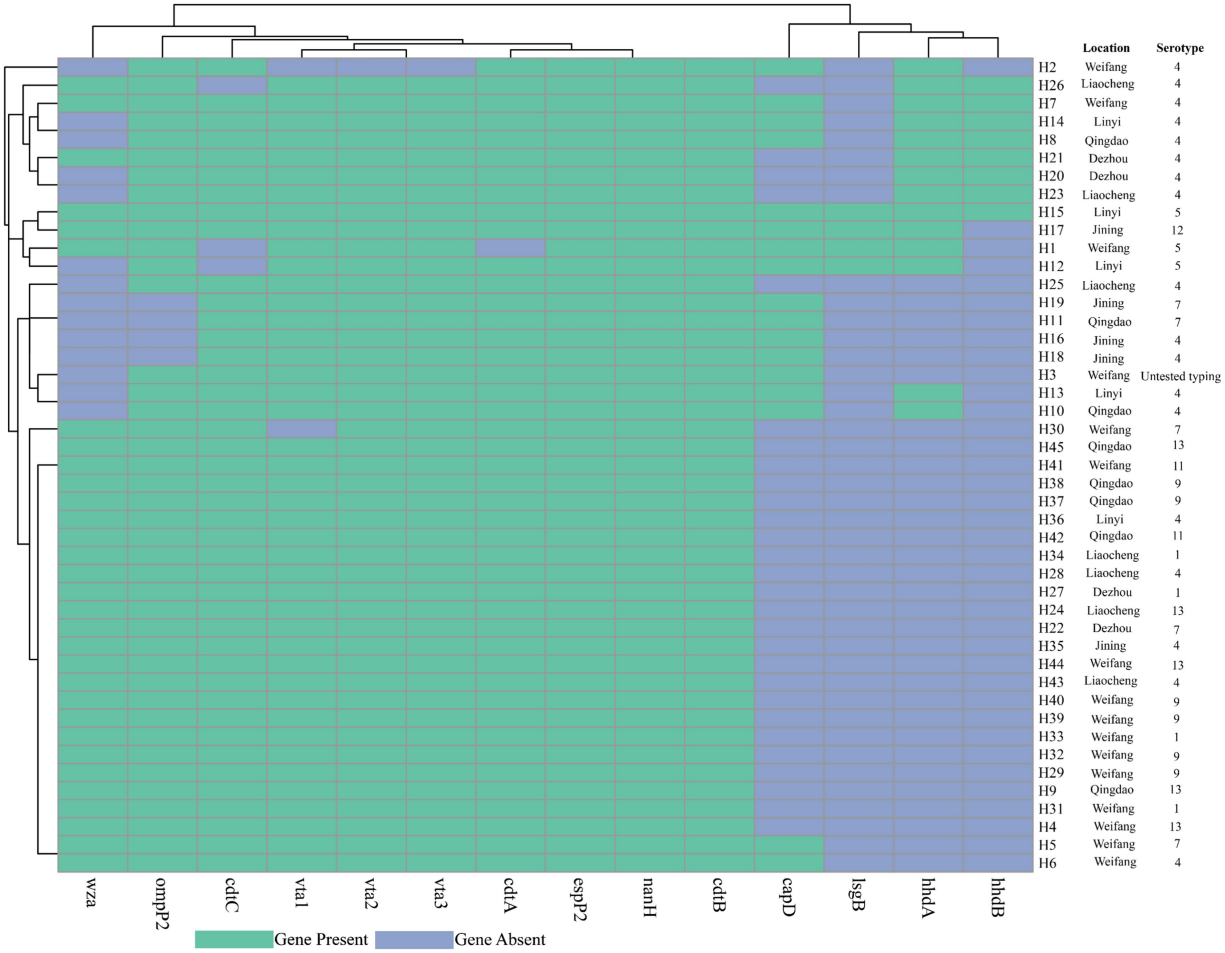
Detection results of *G. parasuis* virulence genes.

The *G. parasuis* virulence genes *nanH*, *cdtB*, and *espP2* were present in all *G. parasuis* isolates. *Vta2*, *vta3*, and *cdtA* were present in 44 *G. parasuis* isolates (97.8%). 43 *G. parasuis* isolates carried *vta1* (95.6%). 42 *G. parasuis* isolates carried *cdtC* (93.3%). 41 isolates carried *ompP2* gene (91.1%). The detection rates of *wza*, *capD*, *hhdA*, *hhdB*, and *lsgB* were 68.9, 33.3, 31.1, 17.8, and 8.9%, respectively (Figure 3). Notably, *lsgB* was only present in serovars 5 and 12 of *G. parasuis* isolates. Moreover, there were no significant differences in the number of virulence genes carried by *G. parasuis* isolates from various regions (*p* ˃ 0.05).

### 3.4 *Glaesserella parasuis* MLST analysis

**Figure 4:**
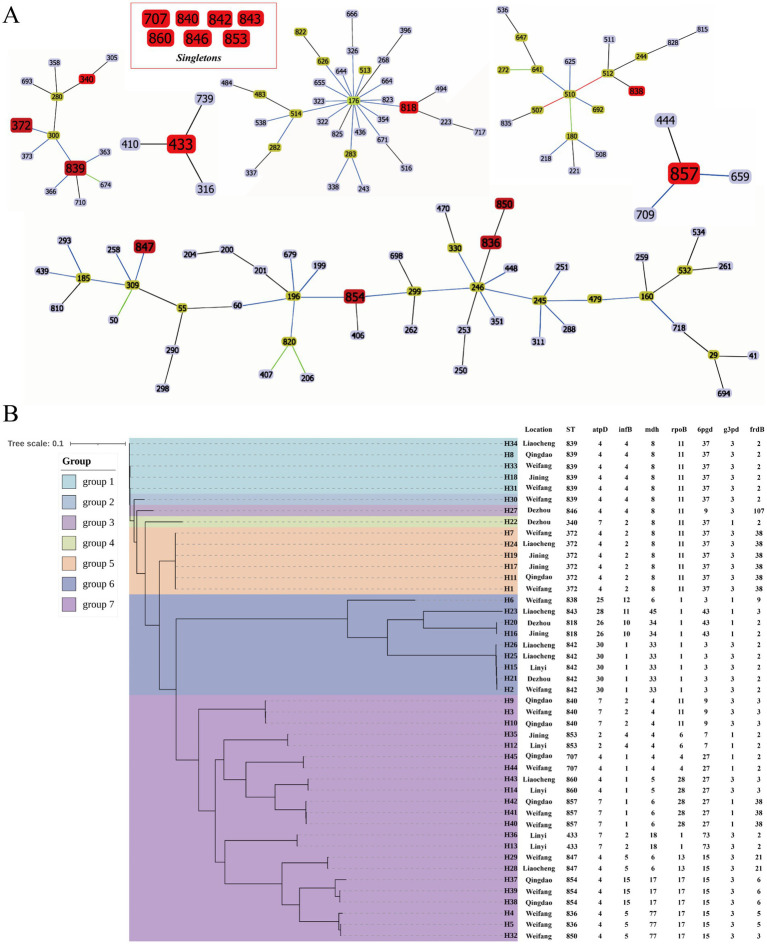
MLST analysis of *G. parasuis*. **(A)** ST cluster analysis of 45 *G. parasuis* and public database using goeBURST. Only clonal clusters or independent classes including our isolates were chosen, with red color representing our isolates. **(B)** A phylogenetic tree consisting of 45 *G. parasuis* isolates generated using the generation of tandem sequences is based on high-quality SNPs. The seven colors on the tree represent the seven groups of analytical identification.

A comparison of the genome sequences of 45 *G. parasuis* isolates with the PubMLST database of *G. parasuis* revealed a total of 18 sequence types (STs), including 5 known STs and 13 newly discovered STs. The newly discovered STs were ST836, ST838, ST839, ST840, ST842, ST843, ST846, ST847, ST850, ST853, ST854, ST857, and ST860. The primary STs in *G. parasuis* isolates from Weifang was ST839 (*n* = 3), whose allele sequence was 4-4-8-11-37-3-2. An analysis of ST clustering among the 45 *G. parasuis* isolates, along with data from the public database using goeBURST software, demonstrated the presence of six clonal clusters and seven distinct classes (Figure 4A). ST836, ST847, ST850, and ST854 were located in the same clonal cluster, and ST372, ST340, and ST839 were located in the same clonal cluster. ST838, ST857, ST433, and ST818 belonged to CC510, CC857, CC433, and CC176, respectively. Phylogenetic analysis of the 18 genotypes of 45 *G. parasuis* isolates revealed that these could be divided into seven clusters, indicating differences in the genetic background of *G. parasuis* (Figure 4B). Moreover, the present study also found that some of the new STs failed to cluster successfully with known STs.

### 3.5 Prophage prediction

**Figure 5:**
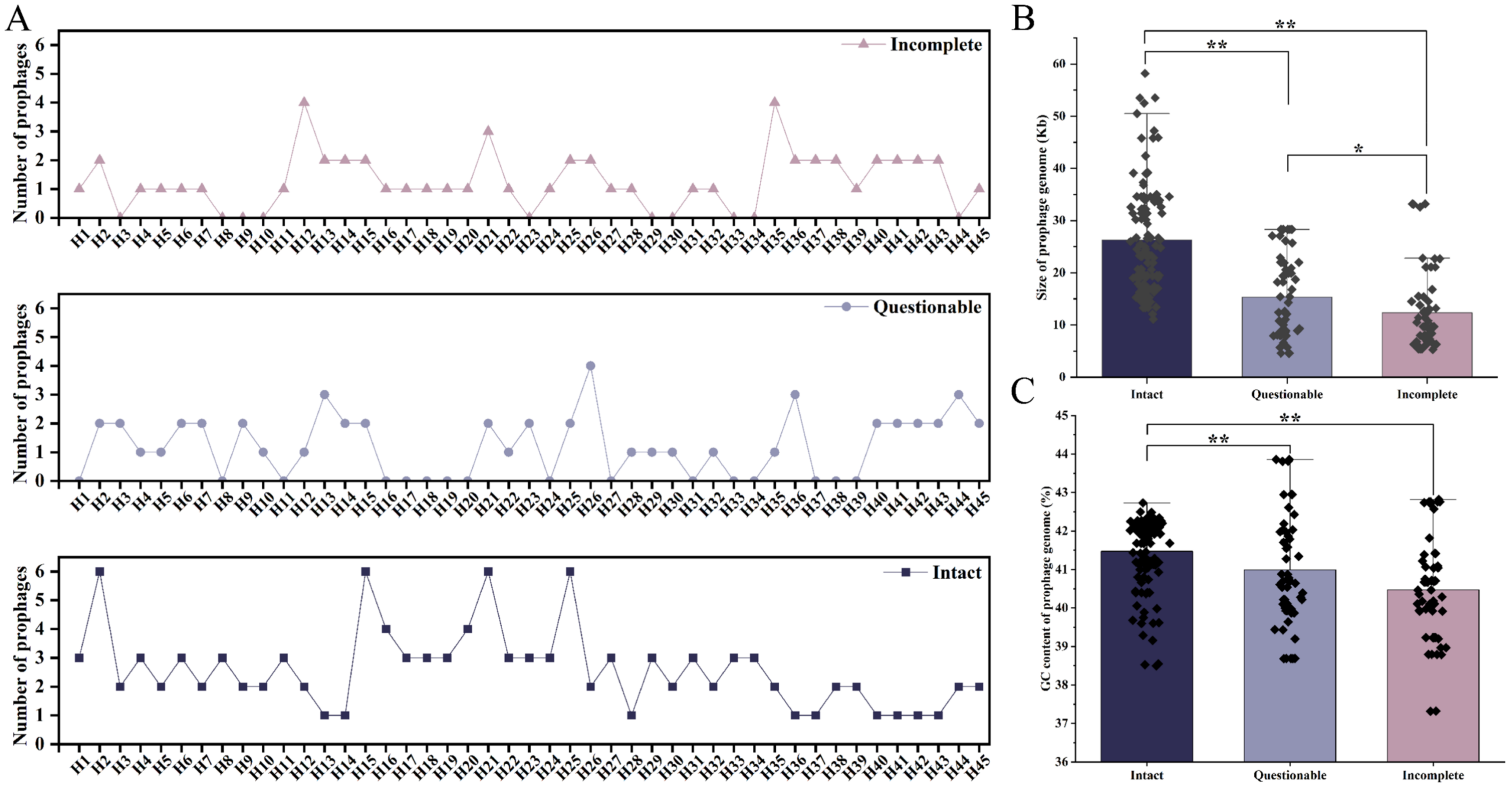
Prediction of prophages. **(A)** Number and type of prophages carried by different *G. parasuis*. **(B)** Genome sizes of different types of prophages. **(C)** GC contents of different types of prophage genomes.

A total of 226 prophages were found in the sequence analysis of 45 *G. parasuis* genomes, including 117 intact, 53 questionable, and 56 incomplete prophages (Figure 5A). The overall analysis of the *G. parasuis* prophage genomes showed that genomes were 4.6–58.2 Kb long, with GC content of 37.3–43.9% (Figures 5B,C). The intact prophages were 11.1–58.2 Kb long, with a GC content of 38.5–42.7%. The questionable prophages were 4.6–28.3 Kb long, with GC content of 38.7–43.9%. The incomplete prophages were 5.3–33.2 Kb long, with a GC content of 37.3–42.8%. Notably, the prophage counts of *G. parasuis* isolates from Weifang were significantly higher than those of *G. parasuis* isolates from the other regions (*p* < 0.01). Additionally, the lengths of incomplete prophage sequences and questionable prophages were significantly shorter than those of strains with intact prophage sequences (*p* < 0.01). The length of incomplete prophage sequences was significantly shorter than that of questionable prophage sequences (*p* < 0.05). The GC content of the incomplete prophage sequences and questionable prophage was significantly lower than that of intact prophage sequences (*p* < 0.01).

### 3.6 *Glaesserella parasuis* pan-genomic analysis

**Figure 6:**
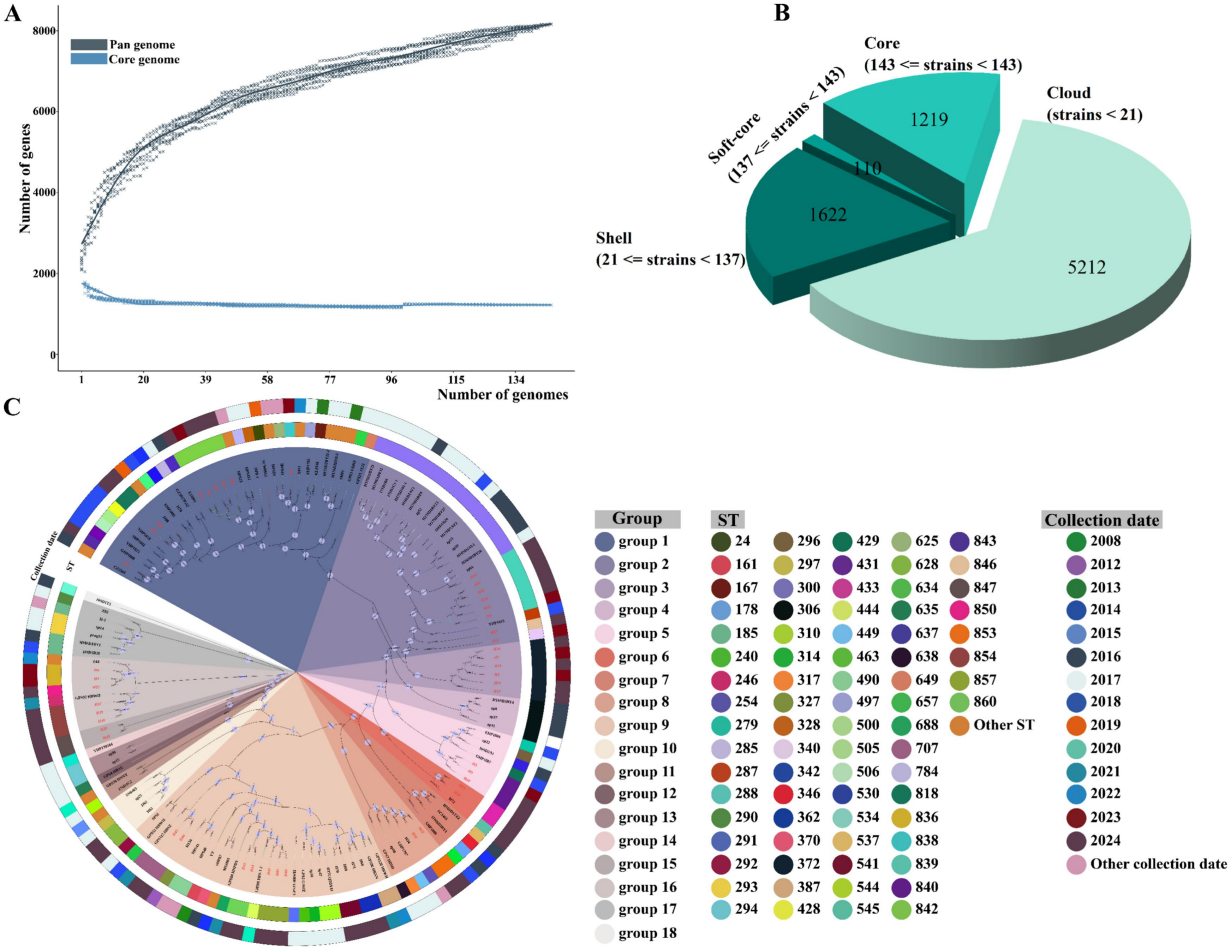
Pan-genomic analysis of *G. parasuis*. **(A)** Accumulation curves of pan-genome and core genome. **(B)** The number of each type of gene contained in the pan-genome and its corresponding number of strains. **(C)** Phylogenetic tree of the core genome of 145 *G. parasuis* isolates. Our isolates are denoted with red italics.

To analyze the relationship between our isolates and public genomic data, we downloaded 100 *G. parasuis* genome sequences from different regions in China between 2008 and 2024 from NCBI at the complete assembly level (Supplementary Table S7). In this study, 145 *G. parasuis* strains had open pan-genomes, and the number of pan-genes increased in direct correlation with the addition of new genomes (Figure 6A). Moreover, the pan-genome of the 145 *G. parasuis* strains contained a total of 8,163 genes, including 1,219 (14.9%) genes in the core genome present of all strains, 110 (1.4%) genes in the soft-core genome, 1,622 (19.9%) genes in the shell genome, and 5,212 (63.9%) genes in the cloud genome (Figure 6B). A phylogenetic tree based on the core genome was constructed, revealing that the strains could be divided into 17 clusters, with the 45 *G. parasuis* isolates in this study distributed across nine of these clusters, indicating the presence of genetic diversity in *G. parasuis* (Figure 6C). Notably, isolates H44 (ST707) and H45 (ST707) were most closely related to strain GPS112 HBSZ (ST707, GenBank: GCA_029767415.1), which was isolated in Hubei Province in 2021. Isolates H37 and H38 were most closely related to strain GPS92 HBWH (GenBank: GCA_029764595.1), which was isolated in Hubei in 2021. However, isolates H37 (ST854) and H38 (ST854) differed from GPS92 HBWH (ST246) in MLST typing, revealing the presence of mutations in two of the seven housekeeping genes for which MLST typing depends. Moreover, ST857 (H40, H41, and H42) and ST860 (H14 and H43) were most closely related to strain LHDR_HPS_1_2 (ST444, GenBank: GCA_015832095.1), which was isolated in Jiangsu in 2020. Except for H5 (ST836) and H32 (ST850), *G. parasuis* isolates of the same STs were found in the same branch. However, H5 and H32 were the most closely related.

## 4 Discussion

Respiratory diseases in swine pose a potential threat to livestock agriculture, with *G. parasuis* being one of the most important microorganisms associated with them. *G. parasuis* is the causative agent of Glässer’s disease, which is characterized by fibrinous polyserositis, meningitis, and pericarditis. Therefore, the isolation and identification of *G. parasuis* have become indispensable gold standards for diagnosing this disease (Wang et al., 2017). The rapid advancement of WGS technology has made the use of bioinformatics tools a crucial approach for investigating the possible genetic diversity of pathogenic *G. parasuis*, ultimately supplying essential data for the clinical management, prevention, and treatment of Glässer’s disease (Mugabi et al., 2023). Our study revealed a prevalence of 17.2% in Shandong Province, which is comparable to the 19.55% reported in Shanxi Province (Yue et al., 2021) but notably lower than both the 43.33% in Zhejiang Province (Xu et al., 2023) and the national prevalence of 27.8% from 2005 to 2019 (Ni et al., 2020). It is worth noting that the national prevalence rate reached 52.1% from 2022 to 2024 (Xu et al., 2025). Our study revealed that the prevalence during autumn and winter was significantly higher than that in spring and summer (*p* < 0.01), which is similar to the results of previous findings. Therefore, it is crucial to strengthen biosecurity measures and possibly implement prophylactic treatments during the cold season. Our study suggests that serovar 4 is predominant in Shandong Province. This is consistent with data reported in China in 2005 (Cai et al., 2005) and 2017 (Wang et al., 2017). However, this appears to contrast with a very recent study which identified serovar 7 as the most prevalent strain in Shandong from 2022 to 2024 (Xu et al., 2025). This difference may be related to the sample size or the sampling time, thus there is an urgent need for continuous molecular monitoring to provide more theoretical basis for disease prevention and control. Moreover, our research indicates that the prevalence rate in Qingdao is higher than in other regions. This regional epidemiological characteristic may be closely associated with local temperature conditions and stocking density. This divergence in positivity and isolation rates may be attributed to the stringent nutritional requirements and specific preservation conditions for *G. parasuis*.

In this study, 45 *G. parasuis* isolates with multiple ARGs carried resistance genes for aminoglycosides, amphenicols, fluoroquinolones, lincomycin, macrolides, sulfonamides, tetracyclines, and *β*-lactams, suggesting that these ARGs have spread widely in the environment. While the detection rates of *gyrA* and *parC* were 100% in *G. parasuis* isolates, the rates of resistance to fluoroquinolones were only 31.1%. This discrepancy underscores that resistance to fluoroquinolones is not conferred by the mere presence of these genes, but is mediated by mutations within the quinolone resistance-determining regions (QRDR), particularly in the *gyrA* and *parC* genes (Kareem et al., 2021). Previous investigations demonstrated that tetracycline resistance has been prevalent among *G. parasuis* isolates, and most resistance genes can be transferred via conjugation and mobile genetic elements (Dayao et al., 2016). Sulfonamide resistance genes (*sul1*, *sul2*, and *sul3*) are commonly associated with bacterial integron systems and conjugative plasmids (Pavelquesi et al., 2021). Amphenicol resistance in *G. parasuis* isolates has been attributed to a novel small plasmid (pHPSF1) carrying *floR*, which is a key contributor to increased resistance (Schwarz et al., 2004; Meunier et al., 2010). Among the β-lactam resistance genes, *tem* is the major resistance gene in *Enterobacteriaceae* (Yang et al., 2018). Our study also found *tem* to be widespread in *G. parasuis* in 48.89% population (22/45) (Guo et al., 2012). The macrolide resistance genes (*erm(A)*, *erm(B)*, and *erm(C)*) showed low frequencies in *G. parasuis* isolates. These genes are responsible for modification of the ribosomal binding site, which is the most important mechanism of resistance to macrolide antimicrobials (Ahmad et al., 2025). Given these elements, the increase in resistance of *G. parasuis* isolates linked to livestock agriculture is not unexpected. The global emergence and spread of MDR bacteria pose an increasing threat to effective antibiotic therapy (Jhalora and Bist, 2025). Among the 45 *G. parasuis* isolates obtained in this study, 31 (68.9%) were resistant to two or more antibiotics and 25 (55.6%) were categorized as MDR strains. This is most likely attributable to the extensive use of antibiotics in livestock farming. In our evaluation of eight antibiotics against *G. parasuis* isolates, we found that amikacin had the highest sensitivity (97.8%, 44/45). This suggests it may be an effective antimicrobial agent for treating *G. parasuis* infection, though further clinical validation is required. Moreover, Comparing ARGs with phenotypic resistance patterns, we found that they showed a “slight” agreement, with the exception of fluoroquinolones. The reason for this outcome may be that we tested for a limited number of resistance genes and antibiotics. Considering the continuing losses to the economy of livestock agriculture caused by *G. parasuis* in Shandong Province, there is a critical need for more effective control measures.

The main virulence genes identified in *G. parasuis* isolates primarily promote pathogenicity by inducing direct damage to host cells and evading host immune defenses (Zhang et al., 2014). Previous studies have demonstrated that many *G. parasuis* isolates harbour detectable virulence genes, including *cdt*, *lsgB*, *espP2*, and *nanH* (Martínez-Moliner et al., 2012; Zhang et al., 2012; Van et al., 2019; Tang et al., 2024). In this study, only serovars 5 and 12 carried *lsgB* in *G. parasuis* isolates, indicating that *lsgB* could be a potential contributor to the enhanced virulence often associated with these serovars (Qi et al., 2021). It has been demonstrated that *vta3* is highly conserved in both strong and weak *G. parasuis* strains, whereas *vta1* and *vta2* are primarily found in pathogenic strains (Wu et al., 2023). In this study, *vta1*, *vta2*, and *vta3* were not found in *G. parasuis* isolate H2, and it carried the least number of virulence genes among all isolates (*n* = 8). Therefore, it can be hypothesized that strain H2 has low pathogenicity compared with the other isolates. *CapD* encodes a protein for polysaccharide biosynthesis associated with the pathogenicity of *G. parasuis*. This gene is often detected in moderately to highly pathogenic strains (Eberle et al., 2020). In this study, we detected *capD* exclusively in serovar 7 isolates. The polysaccharide export protein *wza* has been reported to be highly conserved in the *G. parasuis* genome, which is consistent with the finding that *wza* was present in all serovars strains in this study (Reid and Whitfield, 2005). *OmpP2* can induce the expression of proinflammatory cytokine mRNA in porcine alveolar macrophages (PAMs), and it is among the protective antigens identified in the development of subunit vaccines (Confer and Ayalew, 2013). However, *wza* and *ompP2* were not present in some strains, which may be related to mutations in *G. parasuis* and the horizontal transfer of genes between strains. An earlier study reported that the putative hemolysin operons *hhdA* and *hhdB* are present only in moderately to highly pathogenic strains, and it is a potential virulence factor for *G. parasuis* (Sack and Baltes, 2009). In this study, *hhdA* and *hhdB* were present only in serovars 4, 5, and 12 of *G. parasuis* isolates. Overall, the virulence of *G. parasuis* strains cannot be determined solely by serovar., as strains may acquire pathogenicity islands through horizontal gene transfer, which could potentially alter their pathogenic potential.

In this study, we isolated 45 *G. parasuis* strains from diseased tissues in livestock agriculture in Shandong Province, China, at different time periods, and subjected them to WGS. Sequencing data were annotated and used for both phylogenetic and functional analyses. Their genomes are 2.2–2.7 Mbp in size. Moreover, there was no significant correlation between the number of genes, CDS, and tRNAs of isolates from various regions (*p* ˃ 0.05). The GC content of *G. parasuis* was 39.5–41.0%, which was consistent with previous reports on *G. parasuis* genomes (Mullins et al., 2011). In addition, MLST analysis, which is widely used for transmission pathways and molecular epidemiologic tracing (Mullins et al., 2013), showed that 45 *G. parasuis* isolates had 18 STs, of which 13 newly discovered STs were distributed in different regions of Shandong Province. These results underscore notable geographical variations and highlight considerable genetic diversity among *G. parasuis* isolates. Future research should prioritize functional validation of ST-specific virulence factors and utilize agent-based transmission models to assess their impact on outbreak trajectories.

In this study, all isolates carried intact prophage sequences. This underscores that prophages are significant contributors to the evolution of bacterial hosts (Getz and Maxwell, 2024). Against the backdrop of increasing challenges posed by MRD bacteria and the pressing need for antibiotic alternatives (Dayao et al., 2016), prophages have emerged as a promising therapeutic approach. Research has reported prophages have been successfully isolated from pathogenic bacteria using mitomycin C induction, such as *Vibrio parahaemolyticus* (Qin et al., 2021) and *Clostridium perfringens* (Gervasi et al., 2013). However, there are no reports related to the successful isolation of lytic phages of *G. parasuis*. Therefore, inducing prophages in *G. parasuis* isolates using mitomycin C is a rational and worthwhile strategy for developing phage-based control measures.

The 145 *G. parasuis* strains used in this study were open pan-genomic. This feature may be significantly linked to the acquisition of genes in the livestock agricultural environment, suggesting that the genetic variation of *G. parasuis* species is characterized by considerable diversity and gene transfer (Hurtado et al., 2018). This phenomenon is similar to that observed in species such as *V. parahaemolyticus* (Prithvisagar et al., 2021) and *Escherichia coli* (Yang et al., 2019), which have open pan-genomes. Notably, pan-genomic analyses have been useful for clarifying the evolutionary patterns of pathogenic bacteria. Our study found that *G. parasuis* isolates with the same STs clustered closely within the branches of the core genome evolutionary tree, allowing for a more efficient analysis of the relatedness of strains with the same ST type. Similarly, we found that our isolates were distributed among nine clonal clusters connected to other strains. These connections require further investigation to fully ascertain their pathogenicity. Notably, although our *G. parasuis* strains were isolated from an intensive swine farm in livestock agriculture, our results do not necessarily represent the molecular epidemiology of *G. parasuis* strains in livestock agriculture across China. However, our study effectively demonstrated the epidemiological characteristics of *G. parasuis* in Shandong Province and the utility of systematic genomic analyses to decipher the genetic evolutionary relationship and infection risk posed by *G. parasuis* during livestock agriculture. These findings provide an important foundation for developing regional prevention and control strategies, optimizing livestock biosecurity management, and reducing the economic losses caused by *G. parasuis*.

In this study, we conducted epidemiologic profiling of *G. parasuis* in Shandong Province and comparative genomic analysis of its isolates. Our analyses of the core genome and MLST of these *G. parasuis* isolates enhanced our understanding towards the genetic diversity of pathogenic *G. parasuis*. In addition, we hypothesized relationships between ARGs and AMR phenotypes, and between virulence genes and serovars. Moreover, we also hypothesized that ARGs, AMR phenotypes, virulence genes, and prophages are related to the pathogenicity and defence mechanisms of *G. parasuis*. In future research, we will concentrate on epidemiological investigations and genome continuous monitoring in *G. parasuis*, the evolution and spread of new *G. parasuis* mutant strains in livestock agriculture, and the development of biosafety tools, such as phages, aimed at mitigating the magnitude of epidemics and decreasing the associated risk factors for infection.

## Data Availability

The datasets presented in this study can be found in online repositories. The names of the repository/repositories and accession number(s) can be found in the article/Supplementary material.

## Footnotes

^1^ https://www.addl.purdue.edu/newsletters/1997/spring/dds.shtml

^2^ https://github.com/ablab/spades

^3^ https://github.com/Ecogenomics/CheckM/releases

^4^ https://cge.food.dtu.dk/services/CSIPhylogeny/

^5^ https://itol.embl.de/

^6^ https://phastest.ca/instructions

^7^ https://itol.embl.de/itol_account.cgi
