# Supplementary material for: Epidemiological and comparative genomic analysis of pathogenic Glaesserella parasuis from livestock agriculture in Shandong, China
Source: Front Microbiol. 2025 Oct 8;16:1698342. doi: 10.3389/fmicb.2025.1698342 (PMC12540521; doi:10.3389/fmicb.2025.1698342)
Supplement: Supplementary file 1 [file Supplementary_file_1.zip › Supplementary/Supplementary Figure 1.docx]

| 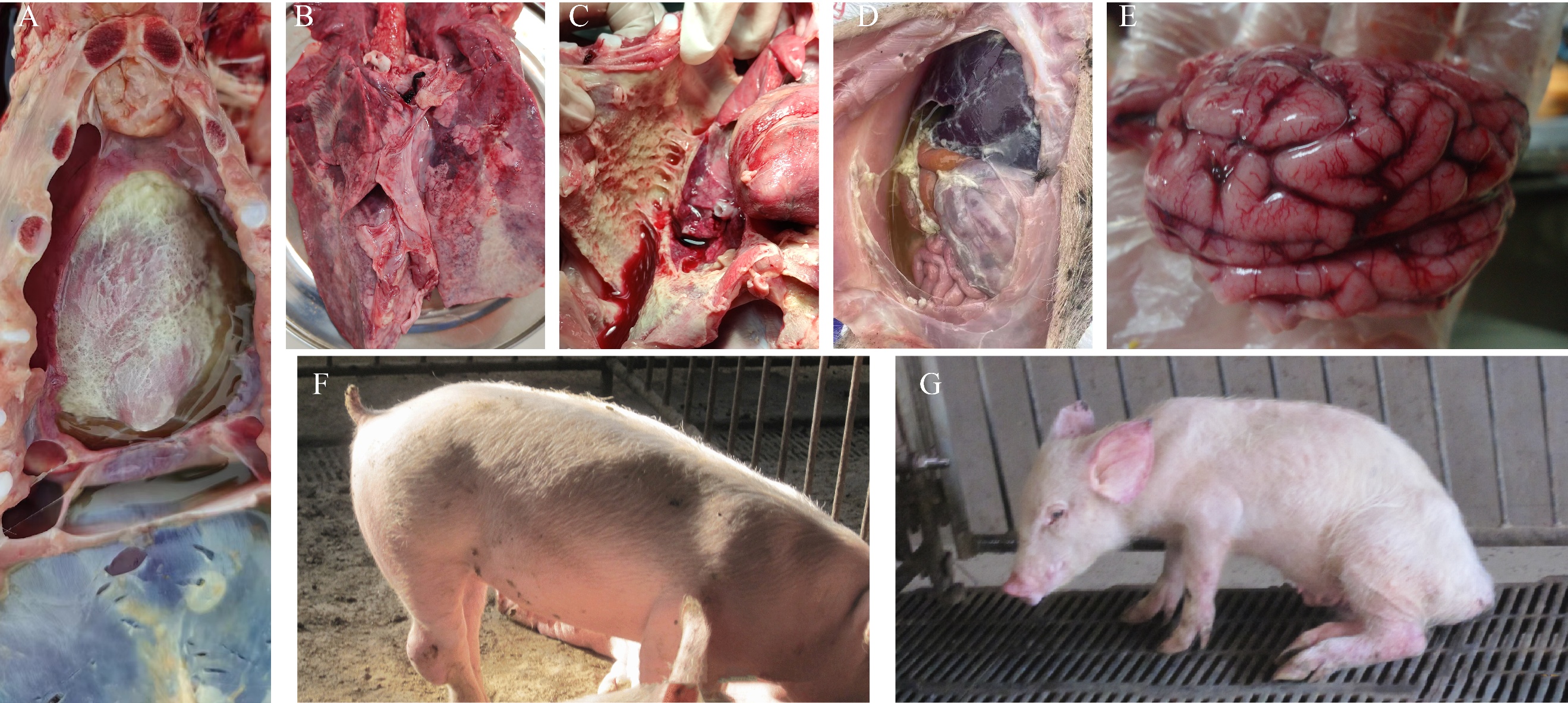 |
| --- |

Figure S1 Necropsy lesions in pigs suspected of being infected with *G. parasuis*. (A). Villous heart; (B). Pulmonary haemorrhage; (C). Thoracic fibrin leakage; (D). Abdominal fibrin leakage; (E). Meningeal congestion and hemorrhage; (F). Gonfiore articolare; (G). Cyanosis of skin
