# Supplementary material for: Epidemiological and comparative genomic analysis of pathogenic Glaesserella parasuis from livestock agriculture in Shandong, China
Source: Front Microbiol. 2025 Oct 8;16:1698342. doi: 10.3389/fmicb.2025.1698342 (PMC12540521; doi:10.3389/fmicb.2025.1698342)
Supplement: Supplementary file 1 [file Supplementary_file_1.zip › Supplementary/Supplementary Figure 2.docx]

| 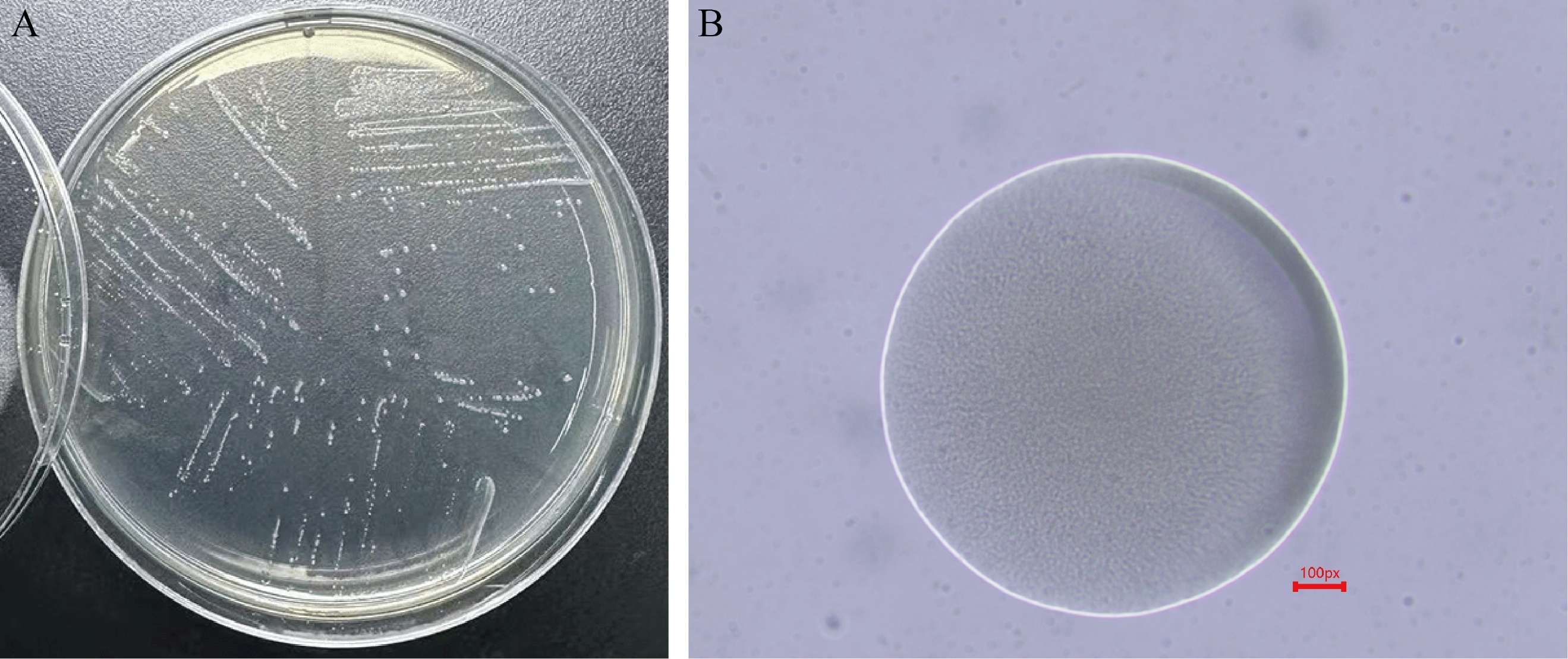 |
| --- |

**Figure S2.** Colony morphology of the isolates. (A) Colony morphology on TSA plates. (B) Colony morphology observed under a light microscope (1000× magnification, oil immersion).
